# Food environment near schools in the largest Brazilian metropolis: analyses and contributions based on census data

**DOI:** 10.1590/0102-311XEN030223

**Published:** 2023-10-09

**Authors:** Maria Alvim Leite, Mayra Figueiredo Barata, Renata Bertazzi Levy

**Affiliations:** 1 Faculdade de Medicina, Universidade de São Paulo, São Paulo, Brasil.; 2 Núcleo de Pesquisas Epidemiológicas em Nutrição e Saúde, Universidade de São Paulo, São Paulo, Brasil.

**Keywords:** Schools, Food Supply, Access to Healthy Foods, Built Environment, Geographic Mapping, Escolas, Abastecimento de Alimentos, Acesso a Alimentos Saudáveis, Ambiente Construído, Mapeamento Geográfico, Escuelas, Abastecimiento de Alimentos, Acceso a Alimentos Saludables, Entorno Construido, Mapeo Geográfico

## Abstract

We aimed to investigate and compare the distribution of establishments that sell food near municipal, state, and private schools in the municipality of São Paulo, Brazil. This cross-sectional, exploratory, and census study was conducted in 3,121 schools. Circular buffers were traced around schools and concentrations or dispersions of food stores (in absolute numbers and densities) were analyzed. A p-trend was calculated to analyze how food stores density behaved as the buffer radius distance increased. Stratified regression models were built to analyze the characteristics of the food environment. Snack bars and street vendors are the most common types of establishments surrounding schools. Some categories of food stores are concentrated (such as candy stores around municipal and private schools, mini markets around municipal schools, and snack bars around private schools), whereas others (such as super and hypermarkets and fruit and vegetable stores) are dispersed around public schools. The food environment around schools shows differences regarding the instance that administers them and private schools have more food stores around them. Poor-quality food environment around schools exposes students to risk factors regarding excessive unhealthy food consumption.

## Resumo

Este estudo transversal, exploratório e censitário realizado em 3.121 escolas buscou investigar e comparar a distribuição de estabelecimentos de venda de alimentos no entorno de escolas municipais, estaduais e particulares da cidade de São Paulo, Brasil. Foram traçados *buffers* circulares ao redor das escolas e analisadas as concentrações ou dispersões de estoques de alimentos (em números absolutos e densidades). Foi calculado o valor de p de tendencia para analisar o comportamento das densidades dos estabelecimentos com o aumento da distância do raio do *buffer*. As características do ambiente alimentar foram analisadas por modelos de regressão estratificada. Lanchonetes e vendedores ambulantes são os tipos de estabelecimentos mais presentes nos entornos das escolas. Algumas categorias de lojas de alimentos estão concentradas (como lojas de doces em torno de escolas municipais e particulares, minimercados em torno de escolas municipais e lanchonetes em torno de escolas particulares), e outras (como super e hipermercados e hortifrútis) estão dispersas em torno de escolas públicas. O ambiente alimentar ao redor das escolas difere de acordo com a instância que as administra, e as particulares têm mais lojas de alimentos nos arredores. O ambiente alimentar de baixa qualidade ao redor das escolas expõe os alunos a fatores de risco para o consumo excessivo de alimentos não saudáveis.

## Resumen

Este estudio transversal, exploratorio y censal realizado en 3.121 escuelas, buscó investigar y comparar la distribución de los establecimientos de venta de alimentos en el entorno de escuelas municipales, estatales y privadas de la ciudad de São Paulo, Brasil. Se trazaron *buffers* circulares alrededor de las escuelas y se analizaron las concentraciones o dispersiones de las existencias de alimentos (en números absolutos y densidades). Se calculó el valor de p de la tendencia para analizar el comportamiento de las densidades de los establecimientos al aumentar la distancia al radio del *buffer*. Las características del entorno alimentario se analizaron por medio de modelos de regresión estratificada. Las cafeterías y los vendedores ambulantes son los tipos de establecimientos más presentes en el entorno de las escuelas. Algunas categorías de tiendas de alimentos están concentradas (como tiendas de dulces alrededor de las escuelas municipales y privadas, minimercados alrededor de escuelas municipales y cafeterías alrededor de escuelas privadas), y otras (como super e hipermercados y tiendas de frutas y hortalizas) están dispersas alrededor de las escuelas públicas. El entorno alimentario alrededor de las escuelas difiere según la instancia que las administra, y hay más tiendas de alimentos en las inmediaciones de las escuelas privadas. El entorno alimentario de baja calidad alrededor de las escuelas expone a los estudiantes a factores de riesgo para el consumo excesivo de alimentos poco saludables.

## Introduction

Food environments are defined as places in which individuals can access food, such as restaurants, supermarkets, street markets, convenience stores, workplaces, schools, and homes ^1^. Schools, communities, and households are the three priority food environments influencing children and adolescents’ food choice and consumption ^2^. The school food environment comprises the spaces, infrastructure, and conditions inside and outside school facilities in which food is available, obtained, purchased, or consumed ^3^.

Facilitated physical access to a kind of food increases its consumption ^4^. In this sense, we highlight the increased availability and consumption of ultra-processed foods ^5^, which are typically ready-to-eat industrial formulations produced with numerous food-derived ingredients and chemical additives ^6^. ultra-processed foods are also rich in sugar, fat, and therefore calories ^7^. They are “ubiquitous”, i.e., they and their advertisements are present in enormous quantities and varieties in various places ^6^. Excessive consumption of ultra-processed foods is a risk factor for overweight in children and adolescents ^8^.

Brazil shows significant differences between public (whether municipal, state, or federal) and private schools concerning food security and nutrition policies. In other words, the instance that manages the school influences the application of policies in these spaces.

In public schools, the most relevant policy to protect the food of children and adolescents is the Brazilian National School Feeding Program (PNAE, acronym in Portuguese), which freely distributes healthy meals (such as rice and beans, vegetables, and a source of protein) and proposes food and nutrition education strategies for students ^9^. The state of São Paulo has an ordinance on which foods can be sold in state public school cafeterias but is not a mandatory regulatory instrument ^10^. In the municipality of São Paulo (the state capital), another ordinance prohibits commercial cafeterias from operating in all schools in the municipal public network ^11^. Private schools in the municipality are not covered by any food and nutrition regulation or policy. Box 1 shows how schools are administered and regulated and the absence of any regulation on the food sold around schools.

**Box 1:** Administration of the most common school types in Brazil in 2020.

MOST COMMON SCHOOL CATEGORIES IN BRAZIL		COVERAGE OF BASIC EDUCATION STUDENTS *	ADMINISTRATION MODE	STUDENT COSTS
Public	Municipal	48.4%	Management and funds from the municipality	Free
	State	32.1%	Management and funds from the state	
Private		18.6%	Private management	Monthly fee

* Source: Anísio Teixeira National Institute of Educational Studies and Research ^44^.

Studies have shown a spatial correlation between schools and establishments that sell food ^12^ ^,^ ^13^ ^,^ ^14^. However, the literature still has little evidence on this issue, especially considering different food and nutrition regulation scenarios. This study aimed to investigate and compare the distribution of establishments that sell food around municipal, state, and private schools in the municipality of São Paulo.

## Materials and methods

### Data and study design

This cross-sectional, exploratory, and census study was conducted with secondary data, using an unit of analysis consisting of municipal and state public and private schools in the municipality of São Paulo, the most extensive Brazilian metropolis and largest city in the Southern Hemisphere with 12.3 million inhabitants ^15^.

School data from the 2017 School Census were extracted from the city hall website (http://geosampa.prefeitura.sp.gov.br). All municipal, state, and private schools that offered elementary school and/or high school were included. Schools that only offered education for youth and adults (n = 35) and federal schools (n = 2) were excluded. The following school data were analyzed: location, administrative dependency (municipal public; state public; and private), size (up to 200 students; from 200 to 500; from 500 to 1,000; and more than 1,000), and type of offered education (only elementary school; elementary and high school; or just high school).

We utilized data from the Human Development Units in which schools are located ^16^. These are urban territorial delimitations that seek to generate homogeneous socioeconomic areas and have a similar concept to neighborhoods. Hence, we will use the nomenclature “neighborhood” hereafter ^17^. The information used was location, Municipal Human Development Index (M-HDI), and total population. M-HDI is a continuous indicator composed of variables of longevity (life expectancy at birth), education (schooling of the adult and young population by school grades), and income (per capita) ^18^. M-HDI was divided into quartiles, in which the first quartile included the most socioeconomically vulnerable neighborhoods.

A 2017 database with information on food-selling establishments from the São Paulo State Finance Department was used to assess food environments. The data used for the establishments consisted of location and type (street vendors, bars, candy stores, bakeries, snack bars, mini-market, super and hypermarkets, fruit and vegetable stores, butcher shops, and fish markets). We sought to analyze the distribution of establishments selling ultra-processed foods around schools which are usually frequented by students. However, super and hypermarkets, fruit and vegetable stores, and butcher and fish markets (which have no such focus) were selected as controls ^19^ ^,^ ^20^. Their presence could show that other reasons, rather than students’ consumption behavior, modulate the installation of food establishments in the school environment.

### Data treatment and analysis

Overall, 3,121 schools and 75,832 food establishments were included in this study. All data were georeferenced. Circular Euclidian buffers with radii of 100, 200, 250, 300, 400, and 500 meters were traced with schools as centroids. The type of establishment within the buffers was computed.

To assess the spatial distribution of establishments in the vicinity of schools, the average densities (establishments per m^2^) of each type of establishment were adjusted (by school size, education offered, M-HDI quartiles, and neighborhood population) for the three categories of schools (municipal, state, and private) within the 100, 200, 300, 400, and 500 meters buffers. Those buffers were built to analyze how densities behaved as the radius distance in the buffer increased since it enabled us to evaluate if the establishments were concentrated or dispersed as their distance increased from schools. A p-trend was calculated for this analysis.

**Figure 1:**
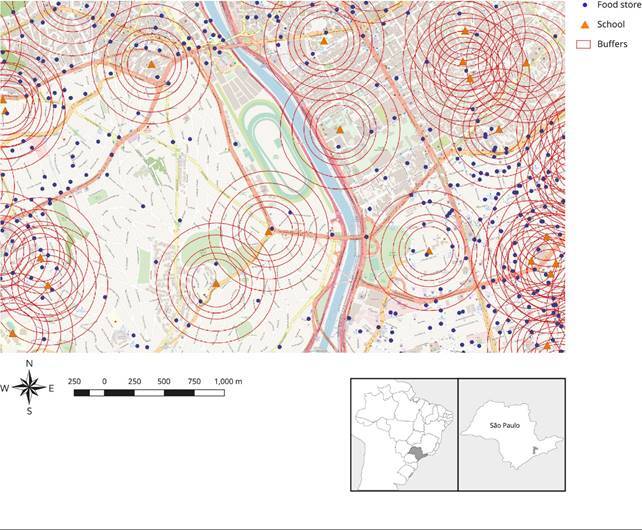
View of the 100, 200, 250, 300, 400, and 500 meters buffers and food outlets around schools in the Pinheiros neighborhood, western region of São Paulo, Brazil, 2017.

To compare the food environment around schools, the average quantity and prevalence (presence of at least one establishment) of types of establishments within a 250m buffer were calculated according to the school administrative dependency. A 250m buffer corresponds to about a 5-minute walking distance children and adolescents usually walk around schools ^21^. Other studies on the food environment around schools used this same distance in their analysis ^22^ ^,^ ^23^ ^,^ ^24^. Figure 1 aids the understanding of this spatial approach.

Regression models were built to analyze the differences between the characteristics of the food environment in the three school categories (municipal, state, and private). Linear regression models considered the administrative dependency of a school, exposure, and the number of establishments as outcomes. Logistic regression models tested the associations between administrative dependencies and presence of establishments in a 250m buffer. In both models, the exposure variable was used as an indicator and the municipal category as reference. They were adjusted for school size, education offered, M-HDI quartiles, and neighborhood population.

Additional analyses were performed to assess the presence of food swamps around the schools. Food swamps refer to areas in which unhealthy food options (such as ultra-processed foods) are readily available and affordable, whereas healthier options are scarce and more expensive ^25^. The surroundings (250m buffers) in which the sum of the number of snack bars, mini markets, and candy stores were greater than or equal to four were considered food swamps ^23^.

QGIS 3.8.1 (https://qgis.org/en/site/) was used to manipulate spatial data. The Geocentric Reference System for the Americas was used as the planimetric reference system (SIRGAS 2000). All analyses were performed using Stata, version 14 (https://www.stata.com). An arbitrary value of p < 0.05 was considered in the models to determine statistical significance.

### Ethical aspects

Ethical approval was unnecessary as this study was conducted with secondary data unrelated to living human subjects or animals.

## Results

Most municipal (65.6%) and state (42.2%) schools were large (more than 1,000 students), whereas most private schools (44%) were small (up to 200 students). Almost 99% of state schools only offered elementary school, whereas 58.9% of state schools offered elementary and high school or only high school. In the private sector, these school categories offered education in about 50% of each. Regarding the socioeconomic level of school addresses, most municipal (41.6%) and state (36.3%) schools were in more vulnerable neighborhoods, whereas most private schools (37.4%) were in less vulnerable neighborhoods. Mean neighborhood populations were similar, with minor variations (around 18,000 inhabitants) (Table 1).

**Table 1:** Description of schools according to their type of administration. São Paulo, Brazil, 2017 (n = 3,121).

Characteristics	Total	Municipal (17.78%)	State (37.04%)	Private (45.18%)
	%	%	%	%
Schools				
Size (students)				
Up to 200	21.1	0.0	3.2	44.0
200-500	20.5	2.5	15.5	31.7
500-1,000	27.8	31.9	39.1	16.9
Over 1,000	30.6	65.6	42.2	7.4
Education offered				
Just elementary school	55.8	98.6	41.1	51.1
Elementary school and high school or just high school	44.9	1.4	58.9	48.9
Neighborhood of the school				
M-HDI (quartiles)				
1	25.3	41.6	36.3	9.7
2	25.3	30.1	25.6	23.1
3	25.3	19.3	22.8	29.8
4	24.2	9.0	15.3	37.4
Population (average)	18,160	17,856	18,099	18,330

M-HDI: Municipal Human Development Index.

According to our analysis of the variation of establishment densities as the area around schools increases, we found that candy stores were concentrated around municipal and private schools (p-trend < 0.05); mini markets around municipal schools (p-trend = 0.04); and snack bars, super and hypermarkets, fruit and vegetable stores, and butcher shops and fish markets around private ones (p-trend < 0.05). We observed a nonsignificant but marginal concentration of bars around municipal schools (p = 0.05). On the other hand, supermarkets and hypermarkets had lower densities around municipal and state schools, i.e., they are more dispersed in radii closer to schools compared to more distant ones (p-trend < 0.05). The same happens between butcher shops and fish markets and state schools (p-trend = 0.01) (Table 2).

**Table 2:** Densities (units per m^2^) of types of food outlets around schools as the area progressively increases. São Paulo, Brazil, 2017.

Establishments/Schools	Radius of 100m	Radius of 200m	Radius of 300m	Radius of 400m	Radius of 500m	p-trend
	Adjusted mean *	Adjusted mean *	Adjusted mean *	Adjusted mean *	Adjusted mean *	
Street vendors						
Municipal	13.3	12.8	12.6	12.9	12.5	0.15
State	13.2	12.6	12.6	12.8	12.6	0.28
Private	15.2	16.0	15.3	15.0	14.4	0.16
Bars						
Municipal	9.2	8.2	8.0	8.0	7.7	0.05
State	7.0	8.3	8.4	8.6	8.4	0.14
Private	10.4	10.8	10.3	9.9	9.6	0.08
Candy store						
Municipal	6.1	6.1	5.9	5.7	5.5	0.01
State	4.7	5.3	5.3	5.3	5.4	0.09
Private	7.8	7.4	6.8	6.5	6.2	< 0.01
Bakeries						
Municipal	6.1	5.9	5.4	5.7	5.5	0.11
State	5.7	5.7	5.9	5.9	5.9	0.06
Private	7.5	8.2	7.7	7.2	6.9	0.19
Snack bars						
Municipal	28.1	29.2	28.5	28.9	28.5	0.75
State	25.9	31.0	31.3	32.1	31.9	0.10
Private	44.5	45.8	42.6	40.2	38.2	0.02
Mini markets						
Municipal	15.1	15.0	13.4	13.7	13.3	0.04
State	12.7	14.3	14.6	14.6	14.4	0.18
Private	16.5	18.3	17.3	16.6	16.1	0.43
Super and hypermarkets						
Municipal	1.0	1.4	1.5	1.7	1.7	0.03
State	1.5	1.5	1.7	1.8	1.8	0.01
Private	2.9	3.1	2.7	2.5	2.4	0.04
Fruit and vegetable stores						
Municipal	4.6	4.6	5.4	6.0	5.8	0.03
State	4.4	5.4	5.7	5.8	6.1	0.03
Private	9.2	8.3	7.4	7.3	7.0	0.01
Butcher shops and fish markets						
Municipal	5.5	3.9	4.0	4.4	4.0	0.28
State	3.0	3.5	3.5	3.7	3.9	0.01
Private	5.9	6.2	5.5	5.2	5.1	0.04

* Adjusted according to school size, offered education, M-HDI (Municipal Human Development Index) quartiles, and neighborhood population.

Snack bars showed the largest quantities within a 250m radius, whereas supermarkets and hypermarkets, the lowest averages. Our adjusted models showed that private schools increased the number of establishments when compared to municipal schools for all establishment types. We found a more accentuated increase for snack bars, i.e., private school increased, on average, the presence of snack bars by three (Table 3).

**Table 3:** Mean, beta coefficient, prevalence, and prevalence ratios (PR) for the categories of establishments within a 250m radius around schools according to the school administrative dependency. São Paulo, Brazil, 2017 (n = 3,121).

Establishments/Schools	Mean (95%CI)	Crude model		Adjusted model *		Prevalence (95%CI)	Crude model		Adjusted model *	
		β	p	β	p		PR	p	PR	p
Street vendors										
Municipal	2.9 (2.7; 3.2)	Reference		Reference		85.1 (81.8; 87.8)	Reference		Reference	
State	2.7 (2.5; 2.8)	-0.26	0.08	-0.01	0.97	79.3 (76.9; 81.6)	0.67	0.01	0.88	0.45
Private	2.7 (2.6; 2.9)	-0.18	0.20	0.54	0.01	80.2 (78.1; 82.2)	0.71	0.01	1.44	0.07
Bars										
Municipal	1.8 (1.6; 2.0)	Reference		Reference		65.6 (61.5; 69.4)	Reference		Reference	
State	1.7 (1.6; 1.9)	-0.06	0.59	0.09	0.49	65.2 (62.4; 67.9)	0.98	0.88	1.27	0.06
Private	1.9 (1.7; 2.0)	0.03	0.76	0.48	< 0.01	66.2 (63.7; 68.7)	1.03	0.78	1.97	< 0.001
Candy store										
Municipal	1.1 (0.9; 1.2)	Reference		Reference		50.8 (46.6; 55.0)	Reference		Reference	
State	1.1 (1.0; 1.2)	0.01	0.88	-0.09	0.43	51.1 (48.2; 54.0)	1.01	0.90	1.11	0.38
Private	1.4 (1.3; 1.5)	0.32	0.00	0.27	0.05	56.3 (53.7; 58.9)	1.25	0.03	1.66	< 0.01
Bakeries										
Municipal	1.3 (1.1; 1.4)	Reference		Reference		56.9 (52.8; 61.0)	Reference		Reference	
State	1.2 (1.1; 1.3)	-0.08	0.35	0.07	0.51	56.6 (53.7; 59.4)	0.99	0.89	1.15	0.26
Private	1.5 (1.4; 1.5)	0.19	0.02	0.52	< 0.001	63.9 (61.4; 66.4)	1.34	0.00	1.96	< 0.001
Snack bars										
Municipal	5.2 (4.6; 5.7)	Reference		Reference		86.9 (83.8; 89.4)	Reference		Reference	
State	6.1 (5.5; 6.6)	0.92	0.08	0.51	0.41	83.0 (80.8; 85.1)	0.74	0.04	1.07	0.69
Private	8.9 (8.2; 9.5)	3.70	0.00	3.08	< 0.001	89.9 (88.2; 91.4)	1.35	0.05	2.57	< 0.001
Mini markets										
Municipal	3.5 (3.2; 3.8)	Reference		Reference		79.6 (76.1; 82.8)	Reference		Reference	
State	3.2 (3.0; 3.4)	-0.33	0.08	0.10	0.65	78.6 (76.1; 80.8)	0.94	0.60	1.18	0.29
Private	2.8 (2.6; 3.0)	-0,72	0,00	0,79	< 0.01	75,7 (73,4; 77,8)	0,80	0,06	1,56	0.02
Super and hypermarkets										
Municipal	0.3 (0.3; 0.4)	Reference		Reference		21.8 (18.6; 25.4)	Reference		Reference	
State	0.3 (0.3; 0.4)	-0.01	0.83	0.02	0.75	21.7 (19.4; 24.2)	0.99	0.97	1.12	0.45
Private	0.6 (0.5; 0.6)	0.22	0.00	0.26	< 0.001	34.0 (31.5; 36.5)	1.85	0.00	2.12	< 0.001
Fruit and vegetable stores										
Municipal	1.4 (1.2; 1.5)	Reference		Reference		58.0 (53.9; 62.1)	Reference		Reference	
State	1.2 (1.1; 1.3)	-0.17	0.07	0.11	0.30	52.7 (49.8; 55.6)	0.81	0.04	1.04	0.77
Private	1.3 (1.2; 1.4)	-0.09	0.32	0.53	< 0.001	55.8 (53.2; 58.4)	0.91	0.38	1.64	< 0.01
Butcher shops and fish markets										
Municipal	0.9 (0.8; 1.1)	Reference		Reference		40.7 (36.7; 44.9)	Reference		Reference	
State	0.8 (0.7; 0.8)	-0.16	0.04	-0.05	0.56	37.8 (35.1; 40.6)	0.88	0.25	1.08	0.53
Private	1.0 (0.9; 1.1)	0.06	0.42	0.40	< 0.01	47.0 (44.4; 49.6)	1.29	0.01	2.14	< 0.001

95%CI: 95% confidence interval.

* Adjusted according to school size, offered education, M-HDI (Municipal Human Development Index) quartiles, and neighborhood population.

As for the presence of establishments around schools, the most common types referred to snack bars and street vendors. Supermarkets and hypermarkets were the least present establishment type. According to our adjusted models, private schools increased the chance of the presence of establishments of all types, except for street vendors. The vicinity of private schools had a 1.6 times greater chance of a snack bar compared to that of municipal schools (Table 3).

We found food swamps in 73.8% of school vicinities. Our analysis of distribution confidence intervals showed a higher prevalence of food swamps in private schools (76.2%) than municipal and state ones (73% and 71.2%, respectively) (data not shown).

## Discussion

In this census study (which analyzed the community food environment around municipal, state, and private schools in one of the largest cities in Latin America), we observed that some categories of food stores are concentrated and others are dispersed around schools. Such territorial associations vary according to the school administrative dependency.

Due to an asymmetrical scenario of regulations, São Paulo has no municipal schools with commercial cafeterias, whereas about 80% of state schools and 100% of private ones do ^26^. Assuming that students have a demand for food that the PNAE fails to provide free of charge, we hypothesized that the absence of commercial cafeterias would boost food sales around municipal schools, as suggested by a study conducted in California (United States) ^27^. Moreover, we observed concentrations of candy stores and mini markets with a varied supply of ultra-processed foods in the vicinity of municipal schools ^19^.

Bars (whose concentration showed a nonsignificant but marginal p-trend in the vicinity of municipal schools) focus on selling alcoholic beverages and snacks for adults. However, we found a variety of available ultra-processed foods (especially candy) on bar counters, which can make them attractive to students ^28^. Although the sale of alcohol is prohibited to the youth, exposure to alcoholic drinks can incentivize consumption, even if illegally. In total, 63% of adolescents enrolled in the 9th grade of elementary schools in São Paulo reported having already tried alcohol ^26^, and the presence of bars close to schools may be associated with higher consumption ^29^.

On the other hand, supermarkets, hypermarkets, and fruit and vegetable shops were dispersed around municipal schools. Although these establishments may offer ultra-processed foods, they do not seem to represent target spots for children and adolescents wandering around their schools. This finding agrees with our hypothesis that the concentration of establishments around schools is not random. The low, disperse density of these establishments around schools points to the formation of a niche-specific commercial area around municipal schools to meet students’ demands.

We found no concentration of any specific establishment around state schools. Instead, we observed a dispersion of control establishments, which may be problematic if we consider the lack of healthy food availability near schools (being more offered in control establishments) ^19^. The vicinity of any school is a living territory in which not only students but also the entire school community circulate (consisting of students’ families, teachers, and school staff).

The vicinity of private schools shows a concentration of candy stores, snack bars, super and hypermarkets, fruit and vegetable stores, and butcher shops and fish markets, i.e., a diffuse pattern of establishment concentration. Furthermore, according to our models for municipal schools, we observed that the presence of a private school increases the number of all types of establishments and the chances of the presence of at least one of all kinds, except for street vendors.

Previous studies conducted with a representative sample of 9th-grade students in the municipality of São Paulo showed that public school students were more economic vulnerable than private ones ^30^. Socioeconomic levels directly imply purchasing power ^31^. Whether for this reason or the movement of people in the region, the surroundings of private schools have a larger supply of all food types.

As mentioned earlier, private schools have no food and nutrition security policy, which makes their students the most exposed - inside and outside schools - to a greater offer of all kinds of foods, especially ultra-processed foods. This can lead to greater consumption and risk of excess weight development ^32^. Previous evidence has suggested that the availability of ultra-processed foods in school cafeterias increases the consumption of these foods ^30^ and that private school students show a higher overweight prevalence (35% against 27% in public ones) ^26^.

We built linear and logistic models to capture slightly different assumptions and dimensions. Linear models assume that more establishments of a type would increase the chance of students visiting it. It can also analyze and compare variations in the quantities of different establishment types within a fixed perimeter. However, when we look at our logistical models, we can hypothesize that the presence of at least one establishment of a certain type would suffice to promote students’ visits. In this case, note that 90% of private schools have at least one snack bar 250m away from them, whereas 85% of municipal schools have at least one street vendor at the same distance. However, both analyses had equivalent results: for all types, the fact that the school is private increases both the number of establishments and the chance of the presence of an establishment (except for street vendors) in a 250m radius. Both approaches find a greater exposure of students from private schools to the risk of consuming unhealthy foods ^33^.

At least two hypotheses can explain the spatial correlation between schools and food outlets in previous studies ^12^ ^,^ ^13^ ^,^ ^14^. In times of face-to-face classes, the flow of people around schools is higher, with children and adolescents coming and going, often accompanied by their guardians. This can encourage the installation of food outlets in these environments given such niche of consumers. Another possibility is that schools are strategically installed in busy places, with several businesses in their surroundings. This strategic location may make more sense for private schools, which are concentrated in wealthier regions. A study conducted in Belo Horizonte (Minas Gerais State), another Brazilian metropolis, found that schools in higher incomes regions had the highest average of all establishments in their surroundings, except for grocery stores and supermarkets ^23^.

Studying in schools whose surroundings expose students to numerous unhealthy foods can harm children and adolescents. Some studies conducted in Brazil have drawn attention to the presence of food swamps around schools ^23^ ^,^ ^34^. São Paulo has been shown to have a higher prevalence of food swamps than Belo Horizonte (73.8% versus 54.6%) ^23^. According to a systematic review ^35^, two studies have found a correlation between the proximity of schools to supermarkets and restaurants with a higher frequency of consumption of chips, sweets, cookies, fried foods, and soft drinks. Another systematic review shows that the sale of food in schools or their immediate vicinity was associated with students’ higher body mass indices and that the availability of healthy food provided by schools significantly decreased students’ odds of obesity ^36^. On the other hand, a systematic scope review claims that food environment around schools are obesogenic, but students feeding practices may not be only related to them since most of their food acquisition and consumption usually happens around family homes ^37^.

Interventions in the school food environment to make it healthier are unable to solve the whole problem but have much potential. By making a wider range of healthy foods available and limiting the supply of ultra-processed foods, food environments comprehensively, persistently, and democratically boost the ability of students and the entire school community to make healthier food choices. Children and adolescents spend many hours in these spaces in which consume between one-third and one-half of their daily meals ^38^. When doing so, they interact with their peers, absorbing and disseminating information and behaviors. Thus, schools configure a crucial space to consulate habits and values ^39^ and are thus fundamental for building healthy eating practices for a lifetime ^40^.

More effective policies aimed at modulating food environments around schools require a detailed characterization of the most at-risk locations and an understanding of social, cultural, demographic, physical, and economic attributes ^41^. It is important to emphasize that policies must economically protect the merchants around the schools in an integrative way either by relocating them to other spaces or providing subsidies to encourage the sale of healthy foods.

This study has limitations due to its cross-sectional design, which implies that the effect measures extracted from it assess associations, rather than causal relationships. It is unfeasible to know whether schools or food outlets were first installed in the neighborhood The fact is that they show territorial associations. Moreover, our use of secondary data sources may lead to inaccurate results, such as underreporting of informal food outlets. However, we believe that this is a nondifferential misclassification that attenuates the effect measures but fails to change their directions. We use Euclidian buffers to determine school territories, which are virtual boundaries in a school neighborhood. We based this territorial cut on previous studies published in peer-reviewed scientific journals ^42^ ^,^ ^43^. Despite its limitations, this census study considers all schools in São Paulo and offers a macro view with unprecedented territory details.

The poor-quality food environment around schools exposes children and adolescents to risk factors for excessive consumption of ultra-processed foods and, consequently, obesity and other detrimental health outcomes. Students’ experiences with the food environment around their schools differ according to the school administrative dependency. Public policies are necessary to regulate food environments around schools and ensure the possibility of equitably choosing adequate and healthy foods.
